# Integrated Determinants of Maternal Healthcare Decisions: A Cross-Sectional Analysis of Predisposing, Enabling, and Illness-Level Factors in Bo District, Sierra Leone

**DOI:** 10.5334/aogh.5148

**Published:** 2026-06-18

**Authors:** Rennie Viah, Rashid Ansumana, Emmanuel Komba Finoh

**Affiliations:** 1 Department of Public Health, College of Health and Medical Sciences, Njala University, Bo City, Sierra Leone

**Keywords:** maternal healthcare-seeking, antenatal care, postnatal care, predisposing factors, enabling factors, illness-level factors, rural–urban disparities, Sierra Leone

## Abstract

*Background:* Maternal and child outcomes in Sierra Leone continue to reflect rural–urban inequities in access to care and timely healthcare-seeking. Understanding how predisposing, enabling, and illness-level determinants interact across settings is essential for explaining these disparities.

*Objectives:* To examine how predisposing, enabling, and illness-level factors influence maternal and child healthcare-seeking decisions in Bo District, and to quantify rural–urban differences in these determinants.

*Methods:* We conducted a facility-based cross-sectional study among 500 women attending antenatal, postnatal, or child health services at an urban tertiary hospital and a rural maternity facility. Descriptive statistics summarized participant characteristics. Rural–urban differences were assessed using Pearson’s chi-square tests, odds ratios with 95% confidence intervals, Mann–Whitney *U* tests for ordinal outcomes, and Cramér’s V to estimate effect sizes.

*Findings:* Predisposing influences on maternal healthcare-seeking were ubiquitous (97.4%), with knowledge and health awareness most frequently reported. Predisposing influences didn’t differ by residence; however, cultural factors were significantly prominent among rural women (*p* = 0.004; Cramér’s V = 0.14). Enabling factors showed the strongest rural–urban differentiation, with small-to-moderate effect sizes. Urban respondents more often reported health insurance coverage, financial stability, proximity to facilities, and transportation availability, whereas rural respondents more frequently reported traditional social, language, and communication support, perceived infrastructure adequacy, preventive service availability, and community engagement. Pregnancy-/childbirth-related complications were common (68.2%) and didn’t differ by residence; however, the perceived influence of illness on care-seeking differed significantly between settings (*p* < 0.001; Cramér’s V ≈ 0.21). For childcare-seeking, knowledge and awareness were foundational across settings, while financial protection and prior healthcare experience exerted greater influence among urban caregivers.

*Conclusions:* Rural–urban differences in maternal and child healthcare-seeking in Bo District appear driven less by awareness or illness occurrence and more by context-specific enabling conditions and differences in how illness is interpreted and acted upon.

## Introduction

Maternal, newborn, and child survival remain a persistent equity challenge in global health, particularly in low-income and fragile health-system settings. Although maternal mortality has declined globally over the past two decades, progress has been uneven, and preventable deaths related to pregnancy and childbirth continue to be concentrated in sub-Saharan Africa, reflecting enduring gaps in timely access to quality antenatal, intrapartum, and postnatal care (PNC) [1–3].

Sierra Leone exemplifies these regional inequities. Despite measurable gains achieved through health-sector reforms and the introduction of free maternal healthcare initiatives, maternal mortality in the country remains among the highest globally, signaling unresolved structural and behavioral barriers to effective service utilization [3, 4]. National analyses consistently demonstrate that maternal healthcare utilization is not determined solely by awareness of services or perceived importance of care. Instead, marked inequalities persist in the adequacy and continuity of antenatal care (ANC), with strong gradients by residence, education, and household wealth [5, 6]. Notably, rural–urban disparities remain evident even when nominal access to services exists, suggesting that deeper social, economic, and health-system constraints shape women’s ability to translate knowledge into timely care-seeking [7]. These patterns are further influenced by sociocultural norms, gendered decision-making structures, and trust in health facilities, which collectively affect when and how women seek care during pregnancy and after childbirth [8].

Conceptually, determinants of maternal and child healthcare-seeking behavior are increasingly understood as a multidimensional decision-making process shaped by interacting social, economic, cultural, and health-system conditions. This study was conceptually informed by Andersen’s behavioral model and its adaptation in maternal healthcare utilization research [9], which distinguishes between predisposing, enabling, and need-related determinants of healthcare use. However, the present study adapted these domains to the Sierra Leonean context by incorporating locally relevant influences such as community engagement, language and communication support, culturally mediated decision-making, and illness-perception factors affecting determinants of maternal, newborn, and child healthcare decisions.

This contextual adaptation reflects the growing recognition that Andersen’s behavioral model is not a rigid framework, but a flexible analytical structure that can be operationalized differently across maternal health settings depending on prevailing social realities, health-system organization, and contextual barriers to care [10]. Maternal health studies applying Andersen-informed frameworks have similarly incorporated setting-specific determinants such as service readiness, communication quality, geographic accessibility, and sociocultural influences to explain variations in maternal healthcare utilization beyond conventional demographic predictors [10, 11].

However, in Sierra Leone, much of the empirical literature remains fragmented. Existing studies often focus on single outcomes, such as the number of ANC contacts, or emphasize isolated barriers like distance or cost, without examining how predisposing, enabling, and illness-level determinants interact within the same population [4, 5]. Moreover, limited attention has been given to how these determinants vary between urban and rural contexts at the subnational level, or how decision-making dynamics shift from maternal care to newborn and child care, where symptom recognition, prior experience, and trust in provider advice may become especially salient [12].

This study advances the maternal, newborn, and child health literature in Sierra Leone in four important ways. First, it applies an integrated determinants framework, predisposing, enabling, and illness-level factors, within a single analytic model of maternal healthcare decision-making. Second, it explicitly compares rural and urban contexts using effect-size estimation alongside statistical testing, allowing clearer interpretation of practical significance beyond statistical associations. Third, it extends the integrated lens to include newborn and child healthcare-seeking decisions, recognizing that household decision rules and thresholds for action may differ between maternal, newborn, and child illness episodes. Finally, by focusing on Bo District and contrasting a tertiary urban hospital with a rural maternity home, the study generates context-specific evidence that captures within-district variation in healthcare decision-making.

Therefore, we aimed to examine how predisposing, enabling, and illness-level factors jointly influence maternal healthcare-seeking decisions in Bo District, Sierra Leone. The primary objective was to assess the association between these integrated determinants and women’s decisions to seek ANC and PNC. The secondary objectives were to (i) compare rural–urban differences in the distribution and influence of predisposing, enabling, and illness-level factors, and (ii) examine how these determinants shape caregivers’ decisions to seek healthcare services for newborns.

## Materials and Methods

### Study design

This study employed a facility-based descriptive cross-sectional design to examine how predisposing, enabling, and illness-level factors influence maternal, newborn, and child healthcare-seeking decisions in Bo District, Sierra Leone. A cross-sectional approach was appropriate for capturing real-time patterns of care-seeking behavior and associated determinants among women actively utilizing maternal and child health services within a defined period.

### Study setting

The study was conducted between January and May 2024 in Bo District, a major population and service center in Sierra Leone’s Southern Province. Two health facilities were selected to represent contrasting healthcare contexts:

- Bo Government Hospital (BGH), a tertiary-level referral hospital located in Bo City, serving urban residents and referrals from surrounding districts.
- Tikonko Maternity Home, a rural primary-level facility serving communities characterized by limited transportation access, lower socioeconomic status, and reduced availability of specialized maternal health services.

The selection of these facilities enabled systematic comparison of maternal, newborn, and child healthcare decision-making across urban and rural settings within the same district.

### Study population

The study population comprised:

- Pregnant women, and
- Mothers of children under 5 years of age,

who attended either facility for ANC, PNC, or routine child health services during the study period.

### Eligibility criteria

Women were eligible for inclusion if they:

- Had resided in the Bo District for at least 6 months,
- Were attending ANC, PNC, or child health services at one of the study facilities,
- Provided voluntary informed consent.

Women were excluded if they were too ill to participate at the time of recruitment, unable to provide informed consent, or had not utilized maternal or child health services during the study period.

### Sampling frame and sampling procedure

Daily sampling frames were constructed using ANC, PNC, and child health attendance registers at each facility. Eligible women present on clinic days were assigned unique identification numbers. Sampling frames were updated daily to include new arrivals and prevent duplicate enrollment.

Participants were selected using simple random sampling, with computer-generated random numbers applied separately within each facility. This approach ensured equal probability of selection and minimized systematic selection bias while maintaining representation across age, parity, education, income, and place of residence.

### Sample size

The minimum sample size was calculated using Cochran’s formula for cross-sectional studies, assuming a 95% confidence level, 5% margin of error, and a conservative prevalence estimate of 50%. This yielded a minimum required sample of 370 participants. To account for potential nonresponse or incomplete data, a 26% adjustment was applied, resulting in a final sample of 500 women, including 294 pregnant women and 206 mothers of children under 5 years.

### Outcome measures

The study outcomes were defined and reflected the variables reported in the Results section.

### Primary outcomes

1. Maternal healthcare-seeking behavior, assessed by the use of ANC and PNC services.
2. Newborn and child healthcare-seeking behavior, assessed through mothers’ reported decisions to seek healthcare services for childhood illnesses or conditions and routine child health beyond PNC.

### Secondary outcomes

- Distribution and relative influence of predisposing factors, enabling factors, and illness-level factors on maternal and newborn healthcare-seeking decisions.
- Rural–urban differences in the prevalence and perceived influence of these factors.

### Definition of key constructs

The study has the following key constructs that need a clearer understanding. Predisposing factors included sociodemographic characteristics, beliefs, attitudes, and culturally mediated influences shaping healthcare decisions before illness recognition or service access. Enabling factors reflected the structural and logistical conditions facilitating or constraining healthcare utilization, including transportation, financial resources, communication support, accessibility of services, and health system responsiveness. Illness-level factors captured symptom recognition, perceived severity of illness, prior healthcare experiences, and interpretations of maternal, newborn, and child danger signs influencing care-seeking urgency. To improve analytical transparency, operational clarification was incorporated for key conceptual categories. For example, “attitudes and beliefs” referred to perceptions regarding trust in healthcare facilities, confidence in formal maternal services, perceived effectiveness of skilled care, and personal beliefs concerning the benefits or limitations of facility-based healthcare. “Cultural factors” referred to culturally mediated influences on decision-making, including family permission structures, traditional childbirth norms, reliance on traditional care pathways, spiritual or customary interpretations of illness, and culturally shaped perceptions regarding the appropriate timing and location for seeking formal maternal, newborn, and child healthcare services. Because participants were permitted multiple responses, the reported frequencies reflected the relative salience of identified influences rather than mutually exclusive categories.

## Data Collection

Data were collected using a structured interviewer-administered questionnaire programmed and deployed through the ONA mobile data collection platform. The questionnaire was pretested at a non-study health facility with similar contextual characteristics to assess clarity, cultural appropriateness, logical flow, and response consistency before formal data collection. To improve participant comprehension and minimize language-related response bias, the tool was translated into Krio and Mende, the predominant local languages spoken within the study communities. Interviews were conducted in private and confidential settings within the health facilities to promote participant comfort, encourage open responses, and reduce the likelihood of social desirability influence during discussions related to maternal and child healthcare experiences. Data collection was conducted by trained enumerators who underwent standardized orientation on research ethics, informed consent procedures, confidentiality, noncoercive interviewing techniques, and electronic data collection protocols. Emphasis was placed on ensuring consistency in question delivery, neutrality during probing, and accurate interpretation of locally contextualized maternal health concepts. Each interview lasted approximately 30–40 minutes. Completed questionnaires were reviewed daily by field supervisors for completeness, consistency, and logical accuracy before secure synchronization and storage within the ONA server. The questionnaire consisted primarily of structured categorical and multiple-response items designed to explore the factors influencing maternal, newborn, and child healthcare-seeking behavior across predisposing, enabling, and illness-level domains. Participants were allowed to select up to three dominant influences affecting their decisions regarding ANC, delivery care, PNC, newborn, and child healthcare utilization. During questionnaire administration, enumerators provided standardized explanations and locally contextualized examples to ensure consistent understanding of the conceptual domains across participants with varying educational and linguistic backgrounds. Responses were subsequently grouped into analytical categories informed by Andersen’s behavioral model and adapted to the sociocultural and health-system realities of Sierra Leone.

### Ethical approval

Ethical approval was obtained from the Njala University Institutional Ethics Review Board. The study adhered to the principles of the Declaration of Helsinki. Participation was voluntary, informed consent was obtained from all participants, and only appropriate and relevant identifier (status, age, sex for under 5 children, education level, marital status, religion, profession/occupation, and tribe) were collected to link participations with the records in the study’s dataset.

### Statistical analysis

Quantitative data were analyzed using SPSS version 26 and R version 4.3. Descriptive statistics were used to summarize participant characteristics and healthcare-seeking behaviors. Categorical variables were presented as frequencies and percentages, while continuous variables were summarized using means and standard deviations, and medians where appropriate.

Rural–urban differences were assessed using Pearson’s chi-square tests for categorical variables and independent t-tests, and Mann–Whitney *U* tests for continuous variables, depending on distributional assumptions.

For selected outcomes, odds ratios (ORs) with 95% confidence intervals (CIs) were calculated to quantify the association between place of residence and reported determinants. Cramér’s V was used to assess effect sizes for chi-square tests and support the interpretation of practical significance.

For ordinal measures of perceived illness influence, the Mann–Whitney *U* test was applied. Statistical significance was assessed at a two-sided alpha level of 0.05.

### Bias and quality control

Selection bias was minimized through random sampling across multiple clinic days and inclusion of both urban and rural facilities. Recall bias was reduced by focusing on recent healthcare experiences. Social desirability bias was mitigated through assurances of confidentiality and neutral question wording. Standardized interviewer training, daily supervision, and routine data checks were implemented to ensure consistency and data quality. Interviewers were recruited locally to match the location and language spoken within the Bo District.

## Results

Table 1 presents the study’s population structure and key sociodemographic characteristics of the 500 participants. Urban respondents predominated, particularly among pregnant women, with an urban-to-rural ratio exceeding 4:1. Mothers of children under 5 and child sex distributions showed more balanced, though still urban-dominant, patterns. Participants were largely young women of reproductive age, with mean ages in the mid-20s for both pregnant women and mothers. Educational attainment was moderate, with over half of respondents reporting secondary education, while income levels were low and highly variable. On average, women reported fewer than two pregnancies, indicating a population largely in early reproductive stages.

**Table 1:** Study population ratios and sociodemographic characteristics of participants (*n* = 500). **A** Study population ratios by residence.

POPULATION CATEGORY	URBAN (BO GOVERNMENT HOSPITAL), *N*	RURAL (TIKONKO MATERNITY HOME), *N*	URBAN: RURAL RATIO
Pregnant women	238	56	4.25:1
Mothers of children under 5	136	70	1.94:1
Children under 5 (total)	137	73	1.94:1
Children under 5—Male	64	38	1.73:1
Children under 5—Female	73	35	2.18:1

**Table:** **B** Sociodemographic characteristics of participants.

CHARACTERISTIC	CATEGORY/MEASURE	*N* (%)	STATISTICAL SUMMARY
**Age of pregnant women (years)**	Mean	–	24.83 ± 4.74 (SE = 0.28; Range: 16–45; 95% CI: 24.29–25.37)
	Median	–	25
	Mode	–	25
**Age of mothers (years)**	Mean	–	24.07 ± 4.34 (SE = 0.30; Range: 17–45; 95% CI: 23.47–24.67)
	Median	–	23
	Mode	–	20
**Educational level**	No formal education	50 (10.0)	–
	Primary education	142 (28.4)	–
	Secondary education	268 (53.6)	–
	Tertiary education	40 (8.0)	–
**Monthly income (SLE)**	Mean	–	335.03 ± 668.49 (SE = 29.90; Range: 0–10,000; 95% CI: 276.29–393.77)
**Pregnancies experienced**	Mean	–	1.83 ± 1.05 (SE = 0.05; Range: 1–7+; 95% CI: 1.74–1.92)

*Note*: Values are presented as *n* (%) for categorical variables and mean ± standard deviation (SD) for continuous variables. CI = confidence interval; SE = standard error; SLE = Sierra Leonean Leone.

Based on recent United Nations and World Bank population reports, the current estimated average number of children per woman in Sierra Leone is at about 3.6–3.8 children per woman. This means fertility in Sierra Leone remains above the global replacement level of 2.1 children per woman, although it has gradually declined over recent decades.

Moreover, 42% of the country’s population is under the age of 15 years, according to UNFPA.

The slightly younger age among mothers compared to pregnant women reflects early childbearing patterns in Sierra Leone. According to the World Bank, among every 1,000 girls ages 15–19 years, 94 gave birth in Sierra Leone in 2023. Many women enter motherhood during adolescence or early adulthood, while older women are more likely to experience repeated pregnancies, increasing the average age among currently pregnant participants. The finding also aligns with the low educational profile of the sample, where limited educational attainment contributes to earlier motherhood and continued reproductive exposure across young adulthood.

The study permitted multiple responses (top three options per participant) for Predisposing Factors. Frequencies and percentages were tallied for each predisposing factor. The study didn’t tally range for Table 2. Subcategories for Any Predisposing Factors were defined and explained to the participants, by enumerators, as any of the following: mother’s level of formal education (secondary education and above), husband or partner education and support, knowledge of maternal services and danger signs, maternal age (15–24 years or 25–34 years), number of previous births (first-time mothers or women with five or more births), place of residence (urban or rural), and maternal health knowledge. The four remaining subcategories were explained during the administration of questionnaires by enumerators to the study participants.

**Table 2:** Predisposing factors influencing antenatal and postnatal care–seeking behavior and decision by residence (multiple responses, *n* = 500).

PREDISPOSING FACTOR	URBAN (BO GOVERNMENT HOSPITAL) *N* (%)	RURAL (TIKONKO MATERNITY HOME) *N* (%)	TOTAL *N* (%)	ODDS RATIO (OR)	95% CI	χ²	*P*-VALUE	CRAMÉR’S V
Any predisposing factor reported	363 (97.1)	124 (98.4)	487 (97.4)	0.53	0.12–2.43	0.69	0.41	0.04
Knowledge and health awareness	305 (75.5)	99 (24.5)	404 (83.0)	Reference	–	–	–	–
Attitudes and beliefs	45 (71.4)	18 (28.6)	63 (12.6)	0.81	0.45–1.47	0.47	0.49	0.03
Cultural factors	3 (33.3)	6 (66.7)	9 (1.8)	0.16	0.04–0.66	10.12	0.004	0.14
Social support and norms	10 (90.9)	1 (9.1)	11 (2.2)	3.25	0.41–25.68	1.38	0.24	0.05
Total predisposing reports	363 (100)	124 (100)	487 (100)	–	–	–	–	–

*Note*: Odds ratios compare the likelihood of reporting each predisposing factor among urban versus rural respondents. OR = odds ratio; CI = confidence interval; χ² = Pearson’s Chi-square test.

Effect size interpretation (Cramér’s V): ≈0.10 = small, ≈0.30 = moderate, ≥0.50 = large association.

As shown in Table 2, nearly all respondents (97.4%) reported at least one predisposing factor influencing their decision to seek ANC or PNC. No statistically significant rural–urban difference was observed for overall predisposing influences, and the corresponding effect size was negligible (Cramér’s V = 0.04), indicating minimal practical variation by residence.

Knowledge and health awareness emerged as one of the dominant maternal preferred predisposing influences, as reported by 75.5% of urban residents and 24.5% rural.

This underscores its central role in maternal healthcare-seeking behavior across both settings. Attitudes and beliefs were reported less frequently and showed no meaningful association with residence, supported by both non-significant statistical results and a very small effect size (Cramér’s V = 0.03).

Cultural factors demonstrated a contrasting statistically significant association with residence (*p* = 0.004) and a small but meaningful effect size (Cramér’s V = 0.14), indicating that cultural considerations were more prominent among rural women when making decisions about maternal care. Although social support and normative influences were infrequently reported, their association with residence was weak (Cramér’s V = 0.05) and not statistically significant.

The combined odds ratios, chi-square tests, and Cramér’s V highlight that while predisposing influences are nearly universal, cultural determinants represent the most context-sensitive predisposing factor, particularly in rural maternal healthcare decision-making.

Table 3 illustrates substantial rural–urban variation in enabling factors influencing maternal healthcare access and decision. Several enabling factors were significantly more likely to be reported by urban respondents, including health insurance coverage, proximity to healthcare facilities, transportation availability, cultural competency of healthcare providers, and supportive social environments. These associations were statistically significant and demonstrated small-to-moderate effect sizes (Cramér’s V = 0.10–0.20), indicating meaningful practical differences beyond statistical significance.

**Table 3:** Enabling factors influencing maternal healthcare access and decision by residence (multiple responses; *n* = 500).

ENABLING FACTOR	URBAN FREQUENCY (*N* = 1,840)	RURAL FREQUENCY (*N* = 627)	TOTAL FREQUENCY	ODDS RATIO (OR)	95% CI	χ²	*P*-VALUE	CRAMÉR’S V
Health insurance coverage	209 (86.36)	33 (13.64)	242	2.31	1.58–3.37	18.96	<0.001	0.20
Financial stability	203 (82.52)	43 (17.48)	246	1.68	1.20–2.37	8.62	0.003	0.13
Proximity to healthcare facilities	162 (85.71)	27 (14.29)	189	2.15	1.41–3.26	12.75	<0.001	0.16
Transportation availability	169 (78.60)	46 (21.40)	215	1.48	1.05–2.08	4.96	0.026	0.10
Cultural competency in healthcare	155 (83.78)	30 (16.22)	185	1.83	1.23–2.74	8.41	0.004	0.13
Supportive social environment	164 (86.32)	26 (13.68)	190	2.26	1.48–3.46	14.28	<0.001	0.17
Geographic accessibility	48 (96.00)	2 (4.00)	50	8.37	2.03–34.54	11.22	0.001	0.15
Cultural and social support (traditional)	4 (16.67)	20 (83.33)	24	0.07	0.02–0.19	39.86	<0.001	0.28
Healthcare infrastructure adequacy	10 (32.26)	21 (67.74)	31	0.16	0.07–0.34	27.45	<0.001	0.23
Language and communication support	9 (31.03)	20 (68.97)	29	0.15	0.07–0.33	27.08	<0.001	0.23
Availability of preventive services	28 (57.14)	21 (42.86)	49	0.45	0.25–0.79	7.06	0.008	0.12
Community awareness and engagement	33 (55.93)	26 (44.07)	59	0.42	0.25–0.71	10.01	0.002	0.14

*Note*: Odds ratios compare the likelihood of reporting each enabling factor among urban versus rural respondents. OR = odds ratio; CI = confidence interval; χ² = Pearson’s Chi-square test.

Effect size interpretation (Cramér’s V): ≈0.10 = small, ≈0.30 = moderate, ≥0.50 = large association.

Geographic accessibility showed the strongest urban association, with urban women far more likely to report physical access to services, although the corresponding effect size remained modest, reflecting the overall rarity of this factor. Financial stability also favored urban respondents, reinforcing the role of economic security in facilitating healthcare access.

In contrast, several enabling factors were significantly more prominent among rural women. Cultural and social support rooted in traditional structures, healthcare infrastructure adequacy, language and communication support, availability of preventive services, and community awareness initiatives were all more frequently reported in rural settings. These factors demonstrated moderate effect sizes (Cramér’s V = 0.14–0.28), highlighting the greater reliance of rural women on community-level, system-dependent, and socially embedded enabling mechanisms.

Overall, the combined use of odds ratios, chi-square tests, and Cramér’s V indicates that enabling factors are not uniformly distributed across settings. Urban access is facilitated primarily through logistical and financial mechanisms, whereas rural access depends more heavily on community engagement, system capacity, and culturally responsive support structures, underscoring the need for context-specific interventions to improve maternal healthcare decision-making and access.

As presented in Table 4A, more than two-thirds of respondents (68.2%) reported experiencing health issues or complications during pregnancy or after childbirth that required medical care. The prevalence of reported complications was similar across urban and rural settings, with no statistically significant association between residence and illness experience (*p* = 0.23). The corresponding effect size was very small (Cramér’s V = 0.05), indicating minimal practical difference in illness occurrence between settings.

**Table 4:** Illness-level factors influencing maternal healthcare-seeking behavior by residence (*n* = 500). **A** Experienced Health Issues or Complications Requiring Care.

RESPONSE CATEGORY	URBAN (BO GOVERNMENT HOSPITAL) *N* (%)	RURAL (TIKONKO MATERNITY HOME) *N* (%)	TOTAL *N* (%)	UNADJUSTED OR	95% CI	χ²	*P*-VALUE	CRAMÉR’S V
Yes	261 (69.8)	80 (63.5)	341 (68.2)	1.33	0.87–2.03	1.44	0.23	0.05
No	113 (30.2)	46 (36.5)	159 (31.8)	Reference	–	–	–	–

OR = odds ratio; CI = confidence interval; *χ*² = Pearson’s Chi-square test.

Effect size interpretation (Cramér’s V): ≈0.10 = small, ≈0.30 = moderate, ≥0.50 = large association.

**Table:** **B** Perceived Influence of Illness-Level Factors on Healthcare-Seeking Decisions (*n* = 341).

INFLUENCE CATEGORY	URBAN *N* (%)	RURAL *N* (%)	TOTAL *N* (%)
Extremely influential	3 (60.0)	2 (40.0)	5 (1.5)
Very influential	93 (90.3)	10 (9.7)	103 (30.2)
Moderately influential	119 (77.3)	35 (22.7)	154 (45.2)
Slightly influential	42 (75.0)	14 (25.0)	56 (16.4)
Not influential	4 (17.4)	19 (82.6)	23 (6.7)

*Note*: Cramér’s V is reported to support practical interpretation of residence-based differences in perceived illness-level influence.

*Statistical test:* Mann–Whitney *U* = 14,165.5.

*p*-value: < 0.001.

Cramér’s V (converted for interpretive consistency): 0.21 (small-to-moderate effect).

Residence-based differences emerged contrastingly in how illness-level factors influenced healthcare-seeking decisions (Table 4B). Urban respondents were more likely to report illness-level factors as very or moderately influential, whereas rural respondents were disproportionately represented among those reporting little or no influence. The Mann–Whitney *U* test confirmed a statistically significant difference in perceived influence by residence (*p* < 0.001), with a small-to-moderate effect size (Cramér’s V = 0.21), suggesting meaningful contextual variation.

These findings indicate that while the occurrence of pregnancy-related complications was broadly similar across urban and rural settings, illness differed substantially. Rural women were more likely to downplay illness severity or delay care-seeking, underscoring the importance of strengthening illness recognition, referral systems, and continuity-of-care mechanisms in rural maternal health programs.

Table 5 presents factors influencing caregivers’ decisions to seek healthcare services for newborns, highlighting clear rural–urban differences. Knowledge and awareness stand as the most frequently reported influence overall, underscoring its foundational role in newborn and child healthcare decision-making across settings.

**Table 5:** Factors influencing the decision to seek healthcare services for children by residence (*n* = 430; multiple responses = 1,425).

FACTOR	URBAN (BO GOVERNMENT HOSPITAL) *N* (%)	RURAL (TIKONKO MATERNITY HOME) *N* (%)	TOTAL *N* (%)	ODDS RATIO (OR)	95% CI	χ²	*P*-VALUE	CRAMÉR’S V
Knowledge and awareness	308 (73.5)	111 (26.5)	419 (29.4)	Reference	–	–	–	–
Cultural beliefs	127 (82.5)	27 (17.5)	154 (10.8)	0.31	0.19–0.50	31.02	<0.001	0.27
Financial resources	149 (81.0)	35 (19.0)	184 (12.9)	0.38	0.24–0.60	27.45	<0.001	0.25
Health insurance coverage	125 (85.6)	21 (14.4)	146 (10.3)	0.28	0.17–0.48	32.18	<0.001	0.27
Symptoms and perceived severity	133 (76.0)	42 (24.0)	175 (12.3)	0.42	0.27–0.66	20.57	<0.001	0.22
Medical advice from health workers	174 (71.9)	68 (28.1)	242 (17.0)	0.66	0.46–0.94	5.10	0.024	0.11
Previous health experiences	63 (60.0)	42 (40.0)	105 (7.4)	0.28	0.18–0.44	27.66	<0.001	0.25

*Note:* Odds ratios compare the likelihood of reporting each factor among urban versus rural respondents. OR = odds ratio; CI = confidence interval; χ² = Pearson’s Chi-square test.

Effect size interpretation (Cramér’s V): ≈0.10 = small, ≈0.30 = moderate, ≥0.50 = large association.

Several factors were significantly more prominent among urban respondents, including cultural beliefs, financial resources, health insurance coverage, perceived symptom severity, and previous health experiences. These associations were statistically significant (*p* < 0.001) and demonstrated small-to-moderate effect sizes (Cramér’s V = 0.22–0.27), indicating meaningful practical differences by residence. Health insurance coverage and cultural beliefs showed the strongest associations, reflecting greater reliance on formal financial protection and belief-guided decision pathways in urban contexts.

Medical advice from health workers was also significantly associated with residence, though the effect size was small (Cramér’s V = 0.11), suggesting that professional guidance influences newborn and child care-seeking in both settings, albeit more consistently in urban areas.

The findings indicate that while knowledge and awareness are universally important, urban mothers are more strongly influenced by financial security, insurance coverage, and prior healthcare experiences when making decisions for newborn and child care. Divergently, the relatively higher rural reliance on symptom recognition and lived experience underscores the need for strengthened community-based education, early recognition of danger signs, and accessible referral pathways to improve timely utilization of newborn and child healthcare.

Table 6 presents patterns of place of childbirth, ANC utilization, and PNC utilization among participants attending the two study facilities. Overall, maternal healthcare utilization was high among women already connected to formal health services, although notable differences in service volume were observed between the urban tertiary hospital and the rural maternity facility.

**Table 6:** Place of childbirth, ANC, and PNC utilization.

**Birth at Health Facility (N = 206)**		
	Bo Government Hospital, *n* (%)	Tikonko Maternity Home, *n* (%)
Yes	135 (65.53)	69 (33.50)
No	1 (0.49)	1 (0.49)
**ANC utilization (N = 294)**		
	Bo Government Hospital, *n* (%)	Tikonko Maternity Home, *n* (%)
Yes	235 (79.93)	54 (18.37)
No	3 (1.02)	2 (0.68)
**PNC utilization (N = 206)**		
	Bo Government Hospital, *n* (%)	Tikonko Maternity Home, *n* (%)
Yes	134 (65.05)	69 (35.50)
No	2 (0.97)	1 (0.49)

Among mothers of children under 5 (N = 206), the overwhelming majority reported delivering at a health facility. Facility-based childbirth was reported by 135 women (65.5%) at BGH and 69 women (33.5%) at Tikonko Maternity Home, while only two respondents across both facilities reported non-facility deliveries. This finding reflects a strong overall preference for skilled delivery care among service-utilizing women within the study population.

ANC utilization among pregnant women (N = 294) was similarly high. Most participants reported utilizing ANC services, including 235 women (79.9%) attending BGH and 54 women (18.4%) attending Tikonko Maternity Home. Only five pregnant women across both facilities reported not utilizing ANC services during the current pregnancy. These findings suggest widespread recognition of the importance of ANC across both urban and rural settings.

PNC utilization among mothers of children under 5 (N = 206) also remained consistently high, with 134 women (65.0%) at BGH and 69 women (33.5%) at Tikonko Maternity Home reporting utilization of PNC services. Only three respondents reported non-utilization of PNC. Although utilization levels appeared favorable within this facility-based sample, the findings should be interpreted cautiously, as women completely disconnected from formal maternal health services were less likely to be captured through facility recruitment.

Collectively, these results indicate that maternal healthcare utilization among facility-attending women in Bo District was generally high across the continuum of care, particularly for ANC attendance and facility-based childbirth.

**Figure 1:**
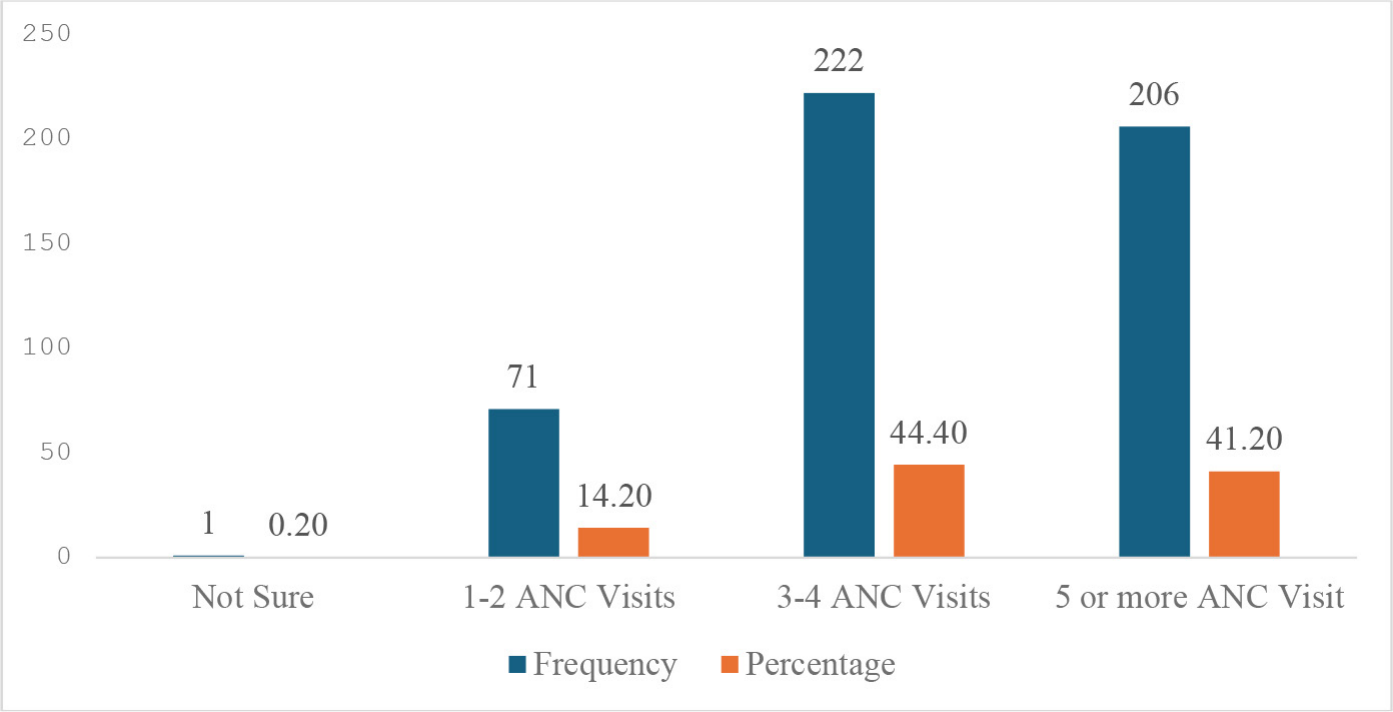
Distribution of antenatal care visits among study participants.

Figure 1 further supports the interpretation of maternal healthcare utilization patterns by showing the frequency of ANC visits among participating women. The figure shows that most respondents reported relatively high engagement with ANC services during pregnancy, although continuity of care remained uneven across participants. Specifically, 222 women (44.4%) reported attending three to four ANC visits, while 206 women (41.2%) attended five or more ANC visits. In contrast, 71 women (14.2%) reported attending only one to two ANC visits, and only one participant (0.2%) was unsure of the number of visits attended. These findings indicate that although ANC contact was generally high among service-utilizing women, a substantial proportion still failed to achieve sustained or optimal engagement with ANC throughout pregnancy.

## Discussion

### Principal findings

This study provides a nuanced, residence-sensitive analysis of maternal, newborn, and child healthcare-seeking behavior in Bo District, Sierra Leone, using a structured predisposing–enabling–illness-level framework supported by effect-size estimation. Four principal findings emerge. First, predisposing influences on care-seeking were nearly universal across settings, with knowledge and health awareness forming a shared baseline determinant. Second, cultural factors, though infrequently reported, were significantly more influential among rural women, indicating context-specific decision thresholds rather than frequency-driven effects. Third, enabling factors demonstrated the strongest rural–urban differentiation, with urban access shaped primarily by financial and logistical resources, and rural access anchored in community, communication, and system readiness. Finally, while illness occurrence was similar across settings, rural women were significantly less likely to perceive illness as a decisive trigger for care, revealing a critical gap between need recognition and action.

Together, these findings indicate that disparities in maternal, newborn, and child care utilization are driven less by differences in awareness or morbidity and more by structural and contextual constraints that condition whether predispositions translate into timely care-seeking.

### Population and sociodemographic context

The urban predominance of pregnant women in this facility-based sample mirrors national patterns of service concentration and referral flow in Sierra Leone, where urban and tertiary facilities attract higher utilization despite broader gains in coverage across rural and urban settings [4, 13]. Maternal health services are largely supported through the Free Health Care Initiative rather than formal nationwide insurance systems, particularly for pregnant women, lactating mothers, and children under 5 in Sierra Leone.

Participants were predominantly young women in early reproductive stages, with mean ages in the mid-20s, consistent with national demographic profiles of ANC and PNC users [5].

Educational attainment clustered at the secondary level, while income was low and highly variable, underscoring persistent economic vulnerability. Recent analyses demonstrate that education and wealth independently predict adequacy of ANC, insurance enrollment, and continuity of maternal services in Sierra Leone [6, 7, 14]. This sociodemographic configuration provides a coherent explanatory backdrop for the dominance of enabling factors observed in subsequent analyses.

### Continuity of maternal healthcare utilization across the care pathway

Maternal healthcare utilization among women already connected to formal services remained generally high across the continuum of care. Most respondents reported facility-based childbirth, ANC utilization, and PNC attendance, reflecting substantial engagement with formal maternal health services within both the urban tertiary hospital and the rural maternity facility. Facility delivery was nearly universal among mothers, with only two respondents across both sites reporting non-facility childbirth. Similarly, ANC and PNC utilization levels remained consistently high, suggesting widespread recognition of the importance of skilled maternal healthcare services across both urban and rural settings.

These findings align with recent national evidence showing improved maternal healthcare utilization in Sierra Leone following implementation of the Free Health Care Initiative and expanded maternal health investments, although important inequalities in continuity and adequacy of care remain persistent [4, 5]. However, the utilization patterns observed in this study should be interpreted cautiously because the facility-based design inherently captured women already connected to healthcare services, potentially underrepresenting women who remain completely excluded from formal maternal healthcare systems.

Importantly, Figure 1 provides deeper insight into the continuity and adequacy of ANC engagement beyond simple service contact. Although ANC attendance was generally high, continuity of care remained uneven among participants. While 41.2% of women reported attending five or more ANC visits and 44.4% attended three to four visits, approximately one in seven women reported attending only one to two ANC visits during pregnancy.

This distinction is critically important in understanding maternal healthcare-seeking behavior in Sierra Leone. Prior studies have shown that women may initiate ANC but fail to maintain recommended contact schedules due to transportation barriers, financial constraints, competing household responsibilities, dissatisfaction with care, perceived symptom improvement, or changing interpretations of pregnancy risk over time [6]. The present findings reinforce the argument that maternal healthcare utilization should not be interpreted solely as binary service use versus non-use, but rather as a continuum involving timing, frequency, continuity, and responsiveness to evolving maternal, newborn, and child health needs.

The observed concentration of service utilization within the urban tertiary facility further reflects ongoing rural–urban asymmetries in healthcare infrastructure, referral pathways, and service availability. Although rural women demonstrated substantial utilization of ANC, PNC, and facility delivery services, the lower rural service volume likely reflects broader structural constraints affecting healthcare access. Therefore, these findings provide important contextual grounding for the enabling and illness-level inequalities identified in subsequent analyses.

### Predisposing factors: knowledge as a shared baseline, culture as a contextual trigger

Knowledge and health awareness emerged as the dominant predisposing influence across both urban and rural settings, reinforcing evidence that information exposure and health literacy are foundational to maternal care-seeking [5]. However, the absence of meaningful rural–urban differences in overall predisposing influences suggests that knowledge alone does not explain observed utilization gaps.

In contrast, cultural factors showed a statistically significant rural association despite low frequency. This finding aligns with recent qualitative evidence from Sierra Leone indicating that cultural legitimacy, social belonging, and trust in maternity spaces can decisively shape care-seeking behavior when choices are constrained [8]. Importantly, this study demonstrates that cultural determinants exert influence not through prevalence but through situational salience, particularly in rural contexts where social norms mediate permission, timing, and perceived appropriateness of seeking formal care.

### Enabling factors: structural drivers of rural–urban inequality

Enabling factors produced the clearest and most consistent rural–urban separation, confirming their central role in translating intention into action. Urban women were significantly more likely to report health insurance coverage, financial stability, proximity to facilities, transportation availability, and supportive social environments, reflecting well-documented urban advantages in service access and continuity [4, 6].

Recent national analyses showed extremely low insurance enrollment among women in Sierra Leone, with strong stratification by residence, education, and employment, reinforcing the plausibility of insurance as a decisive urban enabling mechanism [7, 15]. Even limited exposure to financial protection can reduce perceived risk and delay in care-seeking, particularly for repeat visits and newborn and child care.

Conversely, rural women more frequently identified community-based support, language and communication facilitation, preventive service availability, infrastructure adequacy, and community engagement as enabling influences. Rather than indicating superior rural infrastructure, this pattern suggests that interpersonal communication, system responsiveness, and social mediation become decisive determinants in resource-constrained settings. Contemporary quality-of-care research in Sierra Leone emphasizes that access is not merely geographic but relational, shaped by respectful communication, comprehension, and trust [8]. These findings underscore the need for differentiated access strategies rather than uniform service expansion.

### Illness-level factors: shared risk, divergent responses

More than two-thirds of participants reported pregnancy- or childbirth-related complications requiring care, with no significant rural–urban difference in occurrence. This confirms that clinical risk is broadly shared across settings, consistent with national and regional evidence [7].

However, residence-based differences emerged sharply in the perceived influence of illness on care-seeking. Urban women were significantly more likely to treat illness as a decisive trigger for seeking care, while rural women were overrepresented among those reporting little or no influence. This pattern reflects a well-documented implementation gap: women may recognize symptoms yet delay action due to transport barriers, cost concerns, normalization of danger signs, or uncertainty about service responsiveness [7].

Similar mechanisms have been observed in newborn care, where danger-sign recognition does not consistently translate into timely facility use without supportive enabling conditions [12]. This study extends insight by demonstrating that illness perception itself is contextually conditioned, reinforcing the need for rural interventions that strengthen illness recognition, referral confidence, and continuity of care.

### Newborn care-seeking: financial protection and experience as urban amplifiers

For newborn and child care, knowledge and awareness again formed the foundational influence across settings. However, urban caregivers were significantly more influenced by financial resources, insurance coverage, perceived symptom severity, and previous healthcare experiences, indicating a more system-linked decision pathway. This aligns with evidence that financial protection and prior positive encounters increase confidence in formal care for children [7].

Although rural mothers more frequently relied on symptom recognition and lived experience when making newborn and child healthcare decisions, this pattern may also reflect limited access to formal enabling resources such as transportation, financial protection, continuity of care, and timely referral systems. Consequently, interventions in rural settings should not only strengthen community-based counseling and danger-sign recognition but also address the structural enabling constraints that limit the timely utilization of maternal, newborn, and child healthcare services. Evidence from comparable West African contexts indicates that strengthening caregiver counseling and follow-up substantially improves the timeliness of neonatal care-seeking [12], supporting the relevance of these findings for rural Sierra Leone.

This study makes four novel contributions to the maternal, newborn, and child health literature in low-resource settings. First, it applies a multi-level behavioral framework (predisposing–enabling–illness) with effect-size reporting, allowing distinction between statistical significance and practical relevance, an approach rarely implemented in facility-based studies in Sierra Leone. Second, it demonstrates that predisposing influences are nearly universal, while enabling factors are the primary drivers of rural–urban inequality, reframing intervention priorities away from awareness alone. Third, it reveals that cultural determinants operate as contextual decision triggers rather than high-frequency barriers, particularly in rural maternal care. Finally, it shows that illness occurrence is similar across settings, but illness interpretation and response differ, highlighting a critical leverage point for reducing delays in care-seeking for both mothers and newborns.

### Implications for policy and practice

Although urban women more frequently reported financial protection, transportation availability, and geographic accessibility as enabling influences, these findings likely reflect existing structural advantages rather than unmet priorities. Conversely, the lower reporting of these factors among rural women may indicate constrained access to such enabling resources rather than reduced importance. Therefore, improving the decision to reduce rural–urban inequities in maternal, newborn, and child healthcare utilization will require targeted strengthening of transportation systems, financial protection mechanisms, referral continuity, and physical access to care in rural settings, alongside investments in culturally responsive communication, community engagement, and illness-recognition support. These findings suggest that policy priorities should address both the determinants that women rely upon and the enabling conditions they remain structurally deprived of.

## Conclusion

This study demonstrates that maternal, newborn, and child healthcare-seeking behavior in Sierra Leone is shaped less by whether women *know* the importance of care and more by whether their social, economic, and health-system environments allow that knowledge to be acted upon. Across urban and rural settings, predisposing influences—particularly knowledge and health awareness—were nearly universal, underscoring that information deficits alone no longer explain persistent inequities in service utilization. Instead, the decisive drivers of disparity lie in the unequal distribution of enabling resources and in how illness is interpreted and responded to within different contexts.

The findings show that urban women’s engagement with maternal, newborn, and child services is primarily facilitated by financial protection, physical proximity, transportation, and prior positive interactions with the health system. In contrast, rural women’s care-seeking decisions are more heavily conditioned by community-level support, language and communication, facility readiness, and culturally mediated trust in services. Importantly, pregnancy- and childbirth-related complications were common in both settings, yet rural women were significantly less likely to perceive illness as a compelling trigger for timely care, revealing a critical gap between clinical need and actionable response.

By integrating predisposing, enabling, and illness-level determinants with effect-size estimation, this study advances the understanding of *how* and *where* inequities in maternal, newborn, and child healthcare-seeking emerge. The results highlight that reducing maternal and neonatal morbidity in Sierra Leone will require moving beyond uniform awareness campaigns toward context-specific strategies that strengthen financial protection and service continuity in urban areas while enhancing communication, referral confidence, and system responsiveness in rural communities. Addressing these structural and relational barriers is essential for transforming widespread knowledge into timely, equitable, and life-saving maternal, newborn, and child healthcare utilization.

## Strengths and Limitations

### Strengths

This study has several notable strengths. The relatively large sample size (N = 500) drawn from both urban and rural health facilities enhances the robustness of comparisons within Bo District. The inclusion of maternal, newborn, and child care-seeking decisions provides a more comprehensive view of household health decision-making across the continuum of care. The use of digital data collection tools improved data completeness, reduced entry errors, and enabled real-time quality monitoring. Finally, the application of a structured predisposing–enabling–illness-level framework, complemented by effect-size estimation, strengthens interpretability by distinguishing statistical significance from practical relevance.

### Limitations

Several limitations should be considered when interpreting the findings. The facility-based design may underrepresent women who do not access health services, potentially limiting generalizability beyond service-seeking populations. Analyses of predisposing and enabling factors were based on unadjusted, multiple-response counts, which restrict causal inference and do not account for confounding by sociodemographic characteristics. Because multiple-response items were not mutually exclusive, reported frequencies reflect relative salience rather than independent effects. Some categories had small cell sizes, resulting in wide confidence intervals and requiring cautious interpretation. Finally, self-reported data may be subject to recall and social desirability bias, although standardized interviewer training and confidentiality assurances were used to mitigate these effects.

## Patient and Public Involvement

Patients and members of the public were actively engaged at multiple stages of this study to ensure its relevance, cultural appropriateness, and practical value. Community leaders, facility-based health workers, and women of reproductive age contributed to the refinement of the research focus and the phrasing of survey questions, helping to ensure that the tools reflected locally meaningful experiences, beliefs, and care-seeking realities in both urban and rural settings.

During data collection, participants were informed about the purpose of the study, their voluntary role, and how the findings could inform improvements in maternal, newborn, and child health services. Feedback from participants and community health personnel helped the research team interpret emerging patterns, particularly around cultural influences, enabling barriers, and perceptions of illness severity, grounding the analysis in lived experience rather than abstract assumptions.

Study findings will be shared with participating health facilities and community stakeholders through brief feedback sessions and summary reports to support local dialogue on service quality, access, and responsiveness. While patients and the public were not involved in decisions about study funding or manuscript authorship, their perspectives meaningfully shaped the study design, interpretation of results, and the framing of policy-relevant recommendations.

## Data Availability

Data supporting the findings of this study are available from the Chief Investigator upon reasonable request.

## Data Access Statement

All authors confirm that they had full access to the study data and take responsibility for the integrity and accuracy of the analyses.

## Additional File

The additional file for this article can be found as follows:

Supplementary Appendix A. Structured Questionnaire.

## References

[r16] World Health Organization, United Nations Children’s Fund, United Nations Population Fund, World Bank Group, United Nations Department of Economic and Social Affairs. Trends in maternal mortality 2000–2020 [Internet]. Geneva: World Health Organization; 2023. Accessed May 8, 2026. https://www.who.int/publications/i/item/9789240068759?utm_source=chatgpt.com.

[r12] Shafiq Y, Caviglia M, Juheh Bah Z, et al. Causes of maternal deaths in Sierra Leone from 2016 to 2019: Analysis of districts’ maternal death surveillance and response data. BMJ Open. 2024;14(1):e076256. doi:10.1136/bmjopen-2023-076256. PMC10806740 38216175

[r15] World Bank. Maternal mortality ratio (modeled estimate, per 100,000 live births)—Sierra Leone [Internet]. World Bank. Published 2025. Accessed May 8, 2026. https://data.worldbank.org/indicator/SH.STA.MMRT?locations=SL&utm_source=chatgpt.com.

[r11] Osborne A, Wongnaah FG, Tucker MS. Trends and inequalities in adequate antenatal care coverage among women in Sierra Leone, 2008–2019. Arch Public Health. 2024;82:208. doi:10.1186/s13690-024-01430-1. 39533371 PMC11559174

[r13] Sserwanja Q, Musaba MW, Kamara K, et al. Status of the WHO-recommended antenatal care contacts in Sierra Leone. BMC Health Serv Res. 2022;22:1208. doi:10.1186/s12913-022-08594-y. 36171575 PMC9520872

[r14] Tsawe M, Moto A, Netshivhera T, et al. Inequalities in maternal healthcare use in Sierra Leone. PLoS One. 2022;17(10):e0276102. doi:10.1371/journal.pone.0276102. 36228021 PMC9560049

[r9] Osborne A, Essuman MA, James PB. Determinants of poor access to health care among women of reproductive age in Sierra Leone. BMC Health Serv Res. 2025;25:211. doi:10.1186/s12913-025-12363-y. 39910623 PMC11800412

[r8] Munson O, Shepherd JH, Lewally A, et al. “It shows that I belong”: Birth companionship of choice in a maternity clinic in Freetown, Sierra Leone. BMC Pregnancy Childbirth. 2025;25:618. doi:10.1186/s12884-025-07711-z. 40426127 PMC12107989

[r4] Kabir MR, Khan HTA, Arafat SMY. Adopting Andersen’s behavioral model to identify factors influencing maternal healthcare service utilization. PLoS One. 2021;16(11):e0260622. doi:10.1371/journal.pone.0260502. 34843566 PMC8629289

[r5] Habeeb SA, Abdel Basit IM, Salim AM. Determinants of antenatal care utilization among Saudi females: Application of Andersen’s behavioral model: A cross-sectional study. BMC Pregnancy Childbirth. 2025;25(1):1161. doi:10.1186/s12884-025-08356-8. 41188762 PMC12584230

[r1] Alkhawaldeh A, ALBashtawy M, Rayan A, et al. Application and use of Andersen’s behavioral model as theoretical framework: A systematic literature review from 2012–2021. Iran J Public Health. 2023;52(7):1346–1354. doi:10.18502/ijph.v52i7.13236. 37593505 PMC10430393

[r2] Arthur H, Owusu BA, Osei F, et al. Health seeking behaviour and knowledge on neonatal danger signs among neonatal caregivers in Upper Denkyira East Municipality, Ghana. BMC Pediatr. 2023;23:27. doi:10.1186/s12887-023-04430-2. 38191444 PMC10773034

[r7] Maïga A, Amouzou A, Bagayoko M, et al. Measuring coverage of maternal and child health services using routine health facility data: A Sierra Leone case study. BMC Health Serv Res. 2021;21: 547. doi:10.1186/s12913-021-06529-7. 34511135 PMC8435364

[r6] Kamara IF, Tengbe SM, Issa S. Determinants of quality antenatal care among adolescent girls and women in Sierra Leone: Insights from the 2019 Demographic and Health Survey. Reprod Health. 2026;23:6. doi:10.1186/s12978-025-02236-2. PMC12781750 41372898

[r10] Osborne A, Essuman MA, James PB. Health insurance non-enrollment among women in Sierra Leone. PLoS One. 2025;20(7):e0323538. doi:10.1371/journal.pone.0323538. 40743222 PMC12312921

