# Supplementary material for: Integrated Determinants of Maternal Healthcare Decisions: A Cross-Sectional Analysis of Predisposing, Enabling, and Illness-Level Factors in Bo District, Sierra Leone
Source: Ann Glob Health. 2026 Jun 18;92(1):57. doi: 10.5334/aogh.5148 (PMC13289103; doi:10.5334/aogh.5148)
Supplement: Supplementary Appendix A. — Structured Questionnaire. [file agh-92-1-5148-s1.pdf]

APPENDIX A. STRUCTURED QUESTIONNAIRE

Study Title: "Integrated Determinants of Maternal Healthcare Decisions: A Cross-Sectional Analysis of Predisposing, Enabling, and Illness-Level Factors in Bo District, Sierra Leone"

Target Respondents

Pregnant women and mothers of children under five attending ANC, PNC, or routine child health services at Bo Government Hospital or Tikonko Maternity Home.

SECTION A: FACILITY AND RESPONDENT CLASSIFICATION

1. **Name of health facility**
   - 1 = Bo Government Hospital
   - 2 = Tikonko Maternity Home
2. **Place of residence**
   - 1 = Urban
   - 2 = Rural
3. **Respondent category**
   - 1 = Pregnant woman
   - 2 = Mother of child under five
4. **Has the respondent lived in the Bo District for at least six months?**
   - 1 = Yes
   - 0 = No
   - If no, discontinue interview.

SECTION B: SOCIO-DEMOGRAPHIC CHARACTERISTICS

5. **Age of respondent in completed years: _____**
6. **Marital status**
   - 1 = Single
   - 2 = Married
   - 3 = Cohabiting
   - 4 = Divorced/separated

    - 5 = Widowed

7. **Highest level of education completed**
    - 0 = No formal education
    - 1 = Primary education
    - 2 = Secondary education
    - 3 = Tertiary education
8. **Main occupation**
    - 1 = Student
    - 2 = Farmer
    - 3 = Trader/business
    - 4 = Housewife/unpaid domestic work
    - 5 = Formal employment
    - 6 = Informal employment
    - 7 = Other: specify ______
9. **Average monthly income in Sierra Leonean Leone: _______**
10. **Religion**

- 1 = Christian
- 2 = Muslim
- 3 = Traditional religion
- 4 = Other: specify ______

11. **Ethnic/tribal group: _______**
12. **Number of pregnancies ever experienced: _______**
13. **Number of living children: _______**

SECTION C: MATERNAL HEALTHCARE UTILIZATION

For Pregnant Women

14. **Have you attended antenatal care during this current pregnancy?**

- 1 = Yes

- 0 = No

15. **If yes, how many ANC visits have you attended during this pregnancy?**

- 1 = 1–2 visits
- 2 = 3–4 visits
- 3 = 5 or more visits
- 9 = Not sure

16. **At what month of pregnancy did you first attend ANC?**

- 1 = First trimester
- 2 = Second trimester
- 3 = Third trimester
- 9 = Not sure

For Mothers of Children Under Five

17. **Where did you deliver your most recent child?**

- 1 = Health facility
- 2 = Home
- 3 = Traditional birth attendant's place
- 4 = On the way to the facility
- 5 = Other: specify ______

18. **Did you attend postnatal care after your last delivery?**

- 1 = Yes
- 0 = No

19. **When did you first attend postnatal care after delivery?**

- 1 = Within 24 hours
- 2 = 2–3 days after delivery
- 3 = 4–7 days after delivery
- 4 = After one week
- 9 = Not sure

20. **How many PNC visits did you attend after your last delivery?**

- 1 = One visit
- 2 = Two visits
- 3 = Three or more visits
- 9 = Not sure

SECTION D: PREDISPOSING FACTORS INFLUENCING ANC/PNC CARE-SEEKING

Enumerator instruction:
"Now I will ask about factors that influenced your decision to seek antenatal or postnatal care. Please select up to three factors that most influenced your decision."

21. **Which of the following influenced your decision to seek ANC or PNC? Select up to three.**

Response Option	Code	Analytical Category
I knew ANC/PNC was important for my health and the baby's health	1	Knowledge and health awareness
I knew the pregnancy or newborn danger signs	2	Knowledge and health awareness
Health workers advised me to attend	3	Knowledge and health awareness
My previous pregnancy experience influenced me	4	Attitudes and beliefs
I trusted the health facility or health workers	5	Attitudes and beliefs
I believed facility care was safer than home care	6	Attitudes and beliefs
My husband/partner encouraged or supported me	7	Social support and norms
My family/community encouraged me	8	Social support and norms
Cultural expectations or traditional practices influenced my decision	9	Cultural factors
Family permission or decision-making customs influenced my decision	10	Cultural factors
Preference for traditional care affected my decision	11	Cultural factors
Other: specify _______	12	To be coded after review

SECTION E: ENABLING FACTORS INFLUENCING MATERNAL HEALTHCARE ACCESS

Enumerator instruction:

"Now I will ask about practical conditions that made it easier or harder for you to seek care. Please select all that applied, then identify the three strongest influences."

22. **Which conditions helped or influenced your ability to seek ANC, delivery, or PNC services? Select all that apply.**

**Response Option**	**Code**	**Analytical Category**
I had money to pay for transport or other costs	1	Financial stability
I had health insurance or financial protection	2	Health insurance coverage
The facility was close to my home	3	Proximity to healthcare facilities
Transport was available when I needed it	4	Transportation availability
The road or geographic access was manageable	5	Geographic accessibility
Health workers treated me respectfully and understood my culture	6	Cultural competency in healthcare
I had support from my husband, family, or community	7	Supportive social environment
Traditional/community leaders supported facility care	8	Cultural and social support
The facility had adequate staff, supplies, or space	9	Healthcare infrastructure adequacy
Health workers communicated in a language I understood	10	Language and communication support
Preventive services were available, such as ANC, immunization, health education, or screening	11	Availability of preventive services
Community health workers, volunteers, or outreach workers encouraged me	12	Community awareness and engagement
Other: specify _______	13	To be coded after review

23. **Among the options selected above, which three were the strongest influences?**

- First: _____
- Second: _____

- Third: _____

SECTION F: ILLNESS-LEVEL FACTORS AND CARE-SEEKING

24. **During your current or most recent pregnancy, delivery, or after childbirth, did you experience any health problems or complications that required medical care?**

- 1 = Yes
- 0 = No

25. **If yes, what health problem or complication did you experience? Select all that apply.**

- 1 = Severe abdominal pain
- 2 = Vaginal bleeding
- 3 = Severe headache
- 4 = Swollen feet/face/hands
- 5 = Fever
- 6 = Convulsions/seizures
- 7 = Prolonged labor
- 8 = Weakness/dizziness
- 9 = Infection after delivery
- 10 = Breastfeeding problem
- 11 = Other: specify ______

26. **How much did the illness or complication influence your decision to seek healthcare?**

- 5 = Extremely influential
- 4 = Very influential
- 3 = Moderately influential
- 2 = Slightly influential
- 1 = Not influential

27. **What was the main reason the illness influenced or did not influence your decision to seek care?**

- 1 = I feared the condition was serious

- 2 = I was advised by a health worker
- 3 = I was advised by a family/community member
- 4 = I thought the condition was normal
- 5 = I first tried home or traditional care
- 6 = Lack of money delayed care
- 7 = Lack of transport delayed care
- 8 = Distance delayed care
- 9 = Other: specify ______

SECTION G: NEWBORN/CHILD HEALTHCARE-SEEKING DECISIONS

For mothers of children under five only.

28. **Have you ever sought healthcare for your child because of illness, symptoms, or routine child health needs?**

- 1 = Yes
- 0 = No

29. **Which factors influenced your decision to seek healthcare for your child? Select up to three.**

Response Option	Code	Analytical Category
I knew the child needed care	1	Knowledge and awareness
I knew newborn or child danger signs	2	Knowledge and awareness
I believed the illness was serious	3	Symptoms and perceived severity
Fever, breathing difficulty, poor feeding, diarrhea, convulsion, or weakness made me seek care	4	Symptoms and perceived severity
A health worker advised me	5	Medical advice from health workers
I had money to pay for care or transport	6	Financial resources

I had health insurance or financial protection	7	Health insurance coverage
Previous experience with illness or facility care influenced me	8	Previous health experiences
Cultural beliefs or family customs influenced where or when I sought care	9	Cultural beliefs
Family members or elders advised me to seek or delay care	10	Cultural beliefs
Other: specify _______	11	To be coded after review

30. **Where did you first seek care for the child?**

- 1 = Government hospital
- 2 = Health center/clinic
- 3 = Private clinic
- 4 = Pharmacy/drug seller
- 5 = Traditional healer
- 6 = Home treatment
- 7 = Other: specify ______

31. **How quickly did you seek care after noticing the child's symptoms?**

- 1 = Same day
- 2 = Next day
- 3 = Two or more days later
- 4 = Only when symptoms became worse
- 9 = Not sure

SECTION H: BARRIERS AND DELAYS

32. **Did anything delay or prevent you from seeking ANC, delivery, PNC, or child healthcare?**

- 1 = Yes
- 0 = No

33. **If yes, what delayed or prevented you? Select all that apply.**

- 1 = Lack of money
- 2 = Lack of transport
- 3 = Long distance
- 4 = Bad road condition
- 5 = Needed husband/family permission
- 6 = Fear of poor treatment by health workers
- 7 = Language barrier
- 8 = Cultural or traditional expectations
- 9 = Did not think the condition was serious
- 10 = Facility lacked medicines or staff
- 11 = Other: specify ______

SECTION I: HEALTH SYSTEM EXPERIENCE

34. **How would you describe the attitude of health workers during your visit?**

- 1 = Very respectful
- 2 = Respectful
- 3 = Neutral
- 4 = Disrespectful
- 5 = Very disrespectful

35. **Were services explained to you in a language you understood?**

- 1 = Yes
- 0 = No

36. **Were medicines, supplies, or services available when you visited?**

- 1 = Yes
- 0 = No
- 9 = Not sure

37. **Would you return to this facility for future maternal or child healthcare?**

- 1 = Yes

- 0 = No
- 9 = Not sure

38. **Would you recommend this facility to another pregnant woman or mother?**

- 1 = Yes
- 0 = No
- 9 = Not sure

SECTION J: OPEN-ENDED CONTEXTUAL QUESTIONS

39. **What is the biggest reason women in your community seek care during pregnancy or after childbirth?**

    Response: ____

40. **What is the biggest reason women in your community delay or avoid care during pregnancy or after childbirth?**

    Response: ____

41. **What would help women in your community seek care earlier and more regularly?**

    Response: ____

42. **What would help mothers seek care faster when a newborn or child becomes sick?**

    Response: ____

CODING FRAMEWORK FOR MANUSCRIPT TABLES

Table 1: Socio-demographic characteristics

Covered by Questions 1–13.

Table 2: Predisposing factors

Covered by Question 21.

Coding:

- Knowledge and health awareness = Q21 codes 1–3
- Attitudes and beliefs = Q21 codes 4–6
- Social support and norms = Q21 codes 7–8
- Cultural factors = Q21 codes 9–11
- Any Predisposing Factor Q21 codes 1–11

Table 3: Enabling factors

Covered by Questions 22–23.

Coding:

- Health insurance coverage = Q22 code 2
- Financial stability = Q22 code 1
- Proximity to healthcare facilities = Q22 code 3
- Transportation availability = Q22 code 4
- Geographic accessibility = Q22 code 5
- Cultural competency in healthcare = Q22 code 6
- Supportive social environment = Q22 code 7
- Cultural and social support = Q22 code 8
- Healthcare infrastructure adequacy = Q22 code 9
- Language and communication support = Q22 code 10
- Availability of preventive services = Q22 code 11
- Community awareness and engagement = Q22 code 12

Table 4: Illness-level factors

Covered by Questions 24–27.

Coding:

- Experienced complications = Q24
- Type of complication = Q25
- Perceived influence = Q26
- Reason for care-seeking or delay = Q27

Table 5: Factors influencing newborn/child healthcare-seeking

Covered by Questions 28–31.

Coding:

- Knowledge and awareness = Q29 codes 1–2
- Symptoms and perceived severity = Q29 codes 3–4
- Medical advice from health workers = Q29 code 5

- Financial resources = Q29 code 6
- Health insurance coverage = Q29 code 7
- Previous health experiences = Q29 code 8
- Cultural beliefs = Q29 codes 9–10

Table 6: Place of childbirth, ANC, and PNC utilization

Covered by Questions 14, 17, 18.

Figure 1: Number of ANC visits

Covered by Question 15.
